# Stroke Treatment With PAR-1 Agents to Decrease Hemorrhagic Transformation

**DOI:** 10.3389/fneur.2021.593582

**Published:** 2021-03-15

**Authors:** Patrick D. Lyden, Kent E. Pryor, Jennifer Minigh, Thomas P. Davis, John H. Griffin, Howard Levy, Berislav V. Zlokovic

**Affiliations:** ^1^Department of Physiology and Neuroscience, Keck School of Medicine, Zilkha Neurogenetic Institute, University of Southern California, Los Angeles, CA, United States; ^2^ZZ Biotech LLC, Houston, TX, United States; ^3^inSeption Group LLC, Lansdale, PA, United States; ^4^Department of Medical Pharmacology, University of Arizona College of Medicine, Tucson, AZ, United States; ^5^Department of Molecular Medicine, The Scripps Research Institute, La Jolla, CA, United States; ^6^Howard Levy Consulting LLC, Hopewell, NJ, United States

**Keywords:** hemorrhagic transformation, ischemic stroke, tissue plasminogen activator, intracranial hemorrhage, activated protein C, stroke therapy, thrombectomy, bleeding

## Abstract

Ischemic stroke is the most widespread cause of disability and a leading cause of death in developed countries. To date, the most potent approved treatment for acute stroke is recanalization therapy with thrombolytic drugs such as tissue plasminogen activator (rt-PA or tPA) or endovascular mechanical thrombectomy. Although tPA and thrombectomy are widely available in the United States, it is currently estimated that only 10–20% of stroke patients get tPA treatment, in part due to restrictive selection criteria. Recently, however, tPA and thrombectomy selection criteria have loosened, potentially allowing more patients to qualify. The relatively low rate of treatment may also reflect the perceived risk of brain hemorrhage following treatment with tPA. In translational research and a single patient study, protease activated receptor 1 (PAR-1) targeted therapies given along with thrombolysis and thrombectomy appear to reduce hemorrhagic transformation after recanalization. Such adjuncts may likely enhance the availability of recanalization and encourage more physicians to use the recently expanded selection criteria for applying recanalization therapies. This narrative review discusses stroke therapies, the role of hemorrhagic transformation in producing poor outcomes, and presents the data suggesting that PAR-1 acting agents show promise for decreasing hemorrhagic transformation and improving outcomes.

## Introduction

Each year about 795,000 people in the United States experience a stroke (1). Of all types of stroke, 87% are ischemic (i.e., caused by an interruption of blood supply), 10% are intracerebral hemorrhage strokes (i.e., caused by a ruptured blood vessel), and 3% are subarachnoid hemorrhage strokes (bleeding into the outermost layer of the brain) (2). Ischemic stroke is the most widespread cause of disability and a leading cause of death in developed countries (3).

The most potent treatment for stroke is recanalization, that is, treatment with intravenous thrombolytics, mechanical revascularization (removal of the clot) known as intra-arterial thrombectomy (IAT), or both. Not all patients respond fully to recanalization; therefore, adjunctive cytoprotective treatments are needed and many development efforts are ongoing to overcome the long history of failed neuroprotection trials (likely due to lack of recanalization documentation). Agents acting on the protease activated receptor 1 (PAR-1) exhibit pleiotropic actions on neurons, glia, and cerebral vascular cells, including cytoprotection and anti-inflammation (4). In the RHAPSODY trial, the PAR-1 acting drug 3K3A-APC appeared to reduce hemorrhagic transformation (5).

In 2015, several successful trials proved the efficacy of IAT for acute ischemic stroke with large vessel occlusions (6–10). Then, it was shown that multimodal imaging permits clinicians to select patients for IAT with great success. Recently, the feasibility of combining IAT with a putative cytoprotectant has been shown in 2 trials. The RHAPSODY trial was the first to include IAT in the clinical trial of a cytoprotectant (5). A larger recent trial allowed IAT use, but only in patients with evidence of good collateral flow (11). These results confirm that recanalization may powerfully influence the effect of putative cytoprotectants.

Stroke continues to be a major public health concern despite significant previous research that has produced treatment approaches addressing acute reperfusion and revascularization (12–14), neuronal protection (12), and regeneration of damaged brain tissue (15, 16). All these tactics were based on scientific principles and preclinical data, yet no candidate cytoprotective therapy has successfully entered clinical practice (15). It is now clear that single-action, single-target agents fail to treat stroke because ischemia produces a combination of pathologic pathways proceeding in parallel that damage neural tissue (17).

This narrative review presents a discussion of stroke therapies, the role of hemorrhagic transformation in producing poor outcomes, and presents the data suggesting that PAR-1 acting agents show promise for decreasing hemorrhagic transformation and improving outcomes.

## Stroke Therapy

Recanalization therapy with thrombolytic drugs such as recombinant tissue plasminogen activator (rt-PA or tPA) is the most common treatment for acute stroke. tPA is approved for intravenous administration within 3 h of onset of acute ischemic stroke in the United States and for up to 4.5 h following the stroke in Europe (1, 18). Thrombolytic therapy with intravenous tPA beyond 4.5 h in select subjects with diffusion/fluid attenuated inversion recovery mismatch on magnetic resonance imaging (MRI) is also recommended, but less frequently possible (19). The most widely feared adverse effect of tPA is symptomatic intracranial hemorrhage (SICH; 3–6%); other risks include systemic bleeding, myocardial rupture (when used to treat acute myocardial infarction), and, in rare cases, anaphylaxis, or angioedema (20). Although tPA is widely available in the United States, only 10–20% of stroke patients receive such treatment (21, 22), primarily because patients may present with mild deficits, are beyond 4.5 h after onset, have conditions or concomitant medications that increase bleeding risk, or for other reasons.

Another effective (however, less frequently used) recanalization therapy for stroke is mechanical thrombectomy (7–9, 23). Use of mechanical thrombectomy (with or without tPA) is considered standard-of-care treatment in patients with documented large vessel occlusion, defined as thromboembolic blockage of the distal internal carotid artery, the M1 or proximal M2 portions of the middle cerebral artery, or the proximal anterior cerebral artery. As shown in Table 1, several well-controlled randomized clinical trials showed benefit following combination therapy of thrombectomy and tPA. In some of these trials, however, patients benefited who were ineligible for tPA and were treated with thrombectomy alone. With careful imaging selection, recanalization with thrombolysis or thrombectomy may be successful as late as 16 or 24 h after last known well time (12, 25, 26).

**Table 1:** Summary of mechanical thrombectomy study outcomes.

**Study**	**Percent achieving reperfusion**	**mRS 0–2**	**SICH**	**Mortality**
ESCAPE (7) *N* = 238	72.4%^a^ [31.2%]^b^	53% [29.3%] *p* < 0.001	3.6% [2.7%] *p* = 0.75	10.4% [19.0%] *p* = 0.04
EXTEND-IA (8) *N* = 70	89%^c^ [34%]^c^ *p* < 0.001	71% [40%] *p* = 0.01	0% [6%]	9% [20%]
MR CLEAN (9) *N* = 500	58.7%^a^ [57.5]^b^	32.6% [19.1%] (95% CI: 5.9–21.2)	7.7% [6.4%]	21% [22%]
REVASCAT (10) *N* = 206	65.7%^a^ [Not Reported]	43.7% [28.2%]^e^ (95% CI: 1.1–4.0)	1.9% [1.9%]^e^ *p* = 1.00	18.4% [15.5%]^e^ *p* = 0.60
SWIFT PRIME (23) *N* = 196	82.8%^d^ [40.4%]^d^ *p* < 0.0001	60.2% [35.5%] *p* = 0.0008	1.0% [3.1%] *p* = 0.37	9.2% [12.4%] *p* = 0.50
THRACE (24) *N* = 414	-	-	3 (2%) of 192 ^f^; *p* = 0.71	27 (13%) of 206; *p* = 0.70

*IV, intravenous; mRS, modified Rankin Scale; SICH, symptomatic intracranial hemorrhage; tPA, tissue plasminogen activator. Data are displayed as Mechanical Thrombectomy Arm [tPA-only Control Arm]*.

a *Defined as achieving thrombolysis in cerebral infarction score of 2b or 3*.

b *Defined as achieving modified arterial occlusive lesion score of 2 or 3*.

c *Defined as reperfusion >90% without SICH*.

d *Defined as reperfusion ≥90%*.

e *23 of 103 control subjects did NOT receive IV tPA treatment*.

f *SICH at 24 h*.

Although recent trials suggest that recanalization therapy for stroke offers great promise, there remains a very large unmet need to reduce stroke-related deficit and ensure improved outcomes. First, not all patients treated with thrombolysis or thrombectomy recover full function. Second, the risk of hemorrhage after recanalization therapies dissuades some practitioners from using them. Thus, adjuvant cytoprotectants are needed to complement recanalization therapies in such patients, or to provide improved outcomes in patients unable to receive thrombolytic or thrombectomy therapy. In past clinical trials that did not include mechanical thrombectomy as a treatment option, it is likely that many patients failed to reperfuse; the candidate adjuvant cytoprotectants may therefore have appeared less likely to benefit the patients. In modern clinical stroke trial design, candidate adjuvant therapy is studied in concert with recanalization. In patients with large vessel occlusion, more than 80% receiving mechanical thrombectomy do recanalize. In patients without documented large vessel occlusion, thrombolytic therapy alone is generally sufficient to reperfuse the microvasculature.

## Hemorrhagic Transformation

Hemorrhagic transformation (HT) is a consequence of ischemic blood–brain barrier breakdown that occurs mainly within 2 weeks of ischemic stroke (27). Following an acute stroke, the cerebral vasculature is damaged, which increases the risk for HT.

The presentation of HT includes minor petechial bleeding (hemorrhagic infarct) and large mass-producing hemorrhages (parenchymal hematoma). Intracranial hemorrhages are classified by both imaging characteristics and the presentation of clinical worsening.

Radiologic classification uses the location and extent of hemorrhage to distinguish among hemorrhage subtypes (see Table 2).

**Table 2:** Anatomic descriptions of intracranial hemorrhages according to the heidelberg bleeding classification (28).

**Class**	**Type and description**
**1**	**Hemorrhagic transformation of infarcted brain tissue**
1a	HI1 Scattered small petechia, no mass effect
1b	HI2 Confluent petechia, no mass effect
1c	PH1 Hematoma within infarcted tissue, occupying <30%, no substantive mass effect
**2**	**Intracerebral hemorrhage within and beyond the infarcted brain tissue**
	PH2 Hematoma occupying ≥30% of the infarcted tissue, with obvious mass effect
**3**	**Intracerebral hemorrhage outside the infarcted brain tissue or intracranial-extracerebral hemorrhage**
3a	Parenchymal hematoma remote from infarcted brain tissue
3b	Intraventricular hemorrhage
3c	Subarachnoid hemorrhage
3d	Subdural hemorrhage

*HI, hemorrhagic infarction; PH, parenchymatous hematoma*.

### Symptomatic vs. Asymptomatic Hemorrhagic Transformation

In addition to radiologic classification, intracranial hemorrhages are labeled asymptomatic, or symptomatic based on an accompaniment of observable neurologic decline.

The term SICH was first used by Levy et al. (29). The National Institute of Neurological Disorders and Stroke (NINDS) trials, defined SICH as “any hemorrhagic transformation temporally related to any worsening in neurologic condition (30).”Over the next 2 decades, this definition was recognized as over-inclusive. Other groups such as the Safe Implementation of Thrombolysis in Stroke-Monitoring Study (SITS-MOST) investigators (31), the European Cooperative Acute Stroke Study (ECASS) II and III investigators (18, 32), and the International Stroke Trial-3 (IST-3) investigators (33) have sought more comprehensive definitions of SICH. Widely used definitions are the SITS-MOST and ECASS II:

- SITS-MOST definition of SICH: Local or remote parenchymatous hematoma (PH)-2 with a worsening (i.e., increase of ≥4) on the National Institutes of Health Stroke Scale (NIHSS) score.
- ECASS II definition of SICH: Any intracranial hemorrhage with a clinical worsening (indicated by clinical deterioration or adverse events) or causing a worsening (i.e., increase of ≥4) in NIHSS score.

Asymptomatic intracranial hemorrhage (AICH) does not have a rigorous definition like SICH. In general, it is described as an imaging-documented brain bleed without a concomitant marked deterioration in the patient's neurologic state observable using a neurological rating scale. Thus, the descriptor “asymptomatic” is a misnomer, as the patient very well may exhibit subtle findings, or more robust findings were they to be examined weeks or months later. In many studies, AICH classification is generally not included so that when meta-analyses are performed, those patients with AICH can only be identified as those patients not having SICH. To complicate matters, there are only a handful of studies that specifically enroll subjects with AICH.

### Symptomatic Hemorrhage—Clearly a Detriment

Regardless of how SICH is defined, it is consistently associated with worse clinical outcomes (34, 35). Hao et al. (34) reported that patients with and without SICH differed significantly using the modified Rankin score (mRS) scores (odds ratio: 1.45; 95% confidence interval [CI]: 1.10–1.81), 90-day mortality (higher in patients with SICH [65.3%] vs. without [18.8%]; *p* < 0.001); furthermore, favorable neurological outcome (defined as mRS 0–2) at 90 days was proportionally lower in patients with SICH (8.9%) than without (51.2%) (*p* < 0.001).

### Asymptomatic Hemorrhage—Likely a Detriment as Well

Whether AICH fosters a negative prognosis remains controversial. Some studies confirmed that AICH has a negative effect on functional outcome. Although there is little clinical trial information regarding possible adverse effects of AICH, the limited available evidence indicates it may not be harmless.

In a study by Kent et al. (36), patients with AICH tended toward worse outcomes, even after adjusting for other prognostic variables (odds ratio: 0.69); however, this trend did not reach statistical significance. The investigators cautioned against concluding that AICH are clinically innocuous based on a lack of statistical effect.

Dzialowski et al. (37) used data obtained from the Canadian Alteplase for Stroke Effectiveness Study to investigate the association between HT type and functional outcome. The authors concluded that the likelihood of a poor outcome following thrombolysis was associated with the extent of hemorrhage. The proportion of patients with a good outcome was 41% with no HT, 30% with HI-1, 17% with HI-2, 15% with PH-1, and 7% with PH-2 (*p* < 0.0001). Although HI-1 was not a predictor of outcome, other types of bleeds were after adjusting for covariates: HI-2 (odds ratio: 0.38; 95% CI: 0.17–0.83), PH-1 (odds ratio: 0.32; 95% CI: 0.12–0.80), and PH-2 (odds ratio: 0.14; 95% CI: 0.04–0.48), thereby suggesting that HI grades of hemorrhagic transformation may not be benign.

Park et al. (38) who sought to determine the impact of asymptomatic hemorrhage transformation on the 3-month outcome, found the odds of a worse outcome were increased by a factor of 2 in patients with AICH compared with those without after acute ischemic stroke. The crude and adjusted odds ratios of AICH for an increment of mRS score at 3 months were 2.94 (95% CI: 2.05–4.24) and 1.90 (95% CI: 1.27–2.82), respectively.

Lei et al. (39) examined whether AICH affects risk of stroke recurrence and a long-term poor outcome. Both SICH and AICH post acute ischemic stroke impacted long-term clinical outcomes. Moreover, patients with SICH or AICH suffered a lower survival rate than did patients without HT in the 1st year following stroke (*p* < 0.001). The investigators suggested that AICH should not be considered clinically innocuous.

In acute ischemic stroke patients undergoing thrombectomy, AICH appeared to be associated with high mortality and worse functional outcomes (40). Specifically, AICH appeared to result in lower odds of functional independence (61.9% of patients without AICH and 35.9% with AICH achieved functional independence at the 3-month follow-up; adjusted *p* = 0.117) and higher odds of deaths (35.9% of patients with AICH vs. 11.1% without AICH died; adjusted *p* = 0.015).

Hao et al. (41) reported that in an Asian population, patients with AICH after endovascular treatment had a lower ratio of excellent outcome (odds ratio: 0.53; 95% CI: 0.33–0.84; *p* = 0.007) compared with patients without ICH. According to the researchers: “Considering the relatively higher incidence (33.5%) and negative impacts on functional outcomes in this study, AICH after endovascular treatment may not be innocuous.”

In a recent study, Li et al. (42) evaluated the prevalence of previous chronic cerebral hemorrhage, especially asymptomatic cases, and the associated factors in patients who experienced an acute ischemic stroke. Overall, 9.4% of patients were determined to have had a previous chronic cerebral hemorrhage, with almost half of these being asymptomatic, indicating that previous chronic cerebral hemorrhage is not uncommon in acute ischemic stroke patients. Furthermore, there were no differences in the clinical characteristics of symptomatic vs. asymptomatic previous chronic cerebral hemorrhage, which complicates the detection of asymptomatic hemorrhage, and according to the authors, could increase the risk of re-bleeding.

While AICH may not be associated with acute observable neurologic deterioration, its presence may undermine long-term neurological functions. As the red blood cells in the microbleeds break down over the following days to weeks, neural toxic effects can emerge including heme-induced cerebral inflammation, neuronal apoptosis, and demyelination (43, 44).

Although many studies report the rate of AICH to be ~10% (30, 45, 46), other studies indicate the rate may be as high as 30–40% when using CT scan (24, 34, 47). AICH occurs at a sufficient frequency such that hemorrhages initially presenting as asymptomatic can eventually result in substantial complications, cause an increase in hospital length of stay, lead to poorer long-term outcomes, and incur higher healthcare costs (48); thus, any bleeding, asymptomatic or not, is a concern following stroke.

## APC and APC Analogs

A promising approach for stroke therapy is based on recently discovered biological properties of APC, which is an endogenous plasma protease with multiple properties including antithrombotic action, cytoprotective propensity, and anti-inflammatory activity in the brain and spinal cord (4). Based on known cellular and molecular mechanisms, an APC approach showed promise in experiments consistent with Stroke Therapy Academic Industry Roundtable and other guidelines (49). Wildtype APC shows potent anticoagulant effects along with cytoprotection and reduced inflammation, and anticoagulants often carry an increased risk for serious bleeding. Therefore, protein engineering of APC was undertaken to reduce bleeding risk.

Signaling-selective APC analogs were engineered to retain normal cell-signaling activity (50–53) but to have greatly diminished anticoagulant activity (<10%) (54), thereby reducing *in vivo* risk for bleeding compared with wildtype APC (55–57).

An engineered form of APC, called 3K3A-APC reflecting lysine to alanine substitutions at positions 191, 192, and 193, offers advantages over wildtype APC (4, 53, 58, 59). 3K3A-APC is a 405-residue APC variant engineered to maximize neuroprotective and cytoprotective activities and minimize anticoagulant activity. It was developed by altering factor Va binding exosites (reducing anticoagulation) on APC without modifying the exosites that recognize and bind to the G-protein coupled receptors, protease-activated receptor 1 (PAR-1), and PAR-3. This mutant retains the cytoprotective cell-signaling effects of native (wildtype) APC but has >90% less of the wildtype anticoagulant effects (54). Glycosylation of recombinant 3K3A-APC differs from wildtype APC because it is expressed in Chinese hamster ovary cells.

Anticoagulants do not improve outcome following stroke (60–62). Thus, the residual anticoagulant activity of 3K3A-APC is not responsible for the benefits seen in animal models (63).

## Rationale for APC and APC Analogs in Treating Stroke

APC is an endogenous serine protease with systemic anticoagulant activity as well as cell-signaling actions that convey endothelial stabilizing, anti-inflammatory, and anti-apoptotic activities, and that promote neurogenesis (59, 64–67). APC is normally generated *in vivo* from zymogen protein C through activation by thrombin on the surface of endothelial cells. This activation requires 2 membrane receptors: the thrombomodulin receptor (which binds thrombin) and the endothelial protein C receptor (which binds protein C). The multiple properties of APC should combine in reversing the effects of an ischemic stroke and in protecting ischemic brain tissue from further damage.

**Figure 1:**
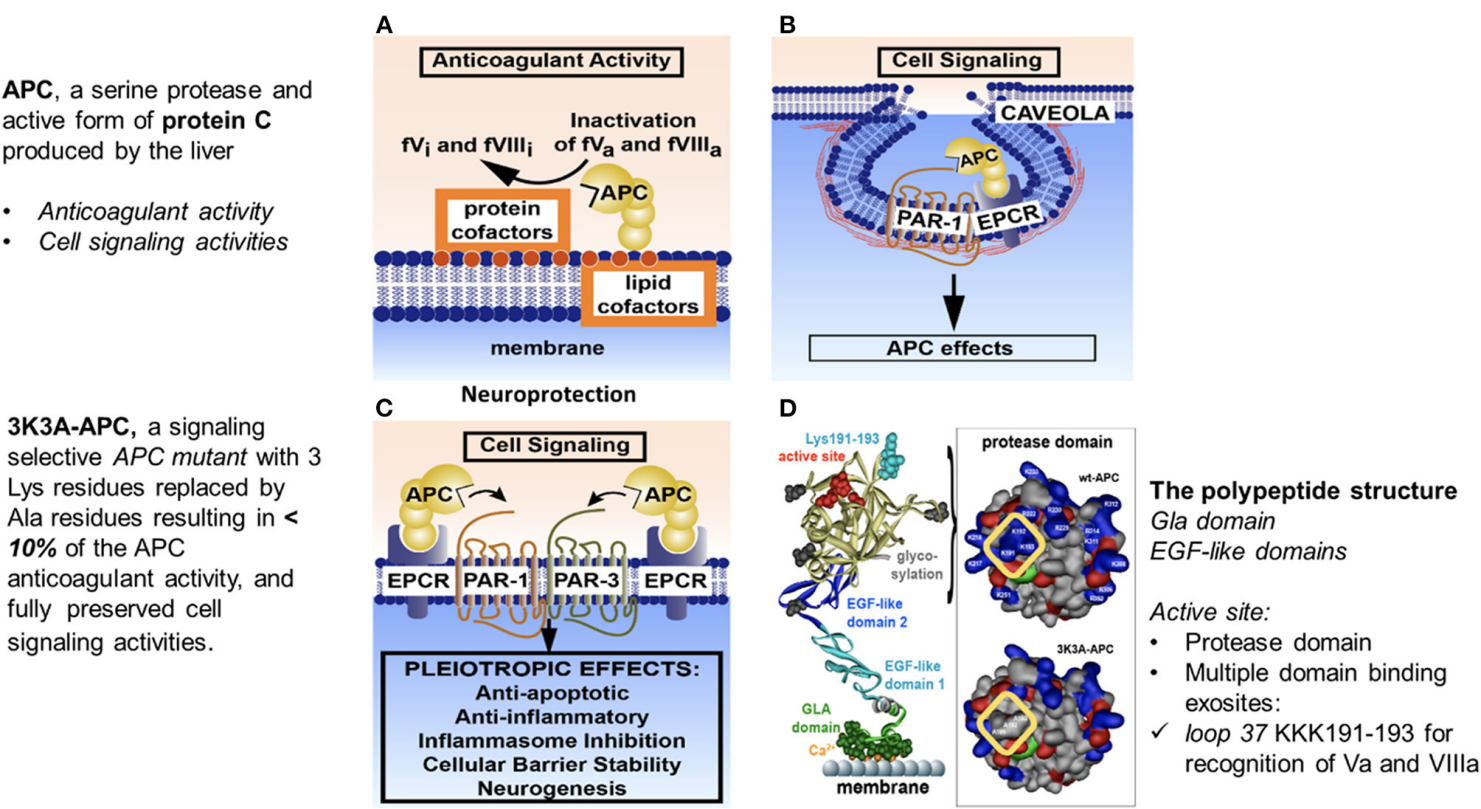
Anticoagulant and cell-signaling pathways of APC and the structure of signaling-selective 3K3A-APC. APC, activated protein C; BBB, blood–brain barrier; EGF, endothelial growth factor; EPCR, endothelial protein C receptor; GLA, gamma-carboxyglutamic acid; PAR, protease-activated receptor. Reprinted from blood, vol. 132(2), Griffin et al. (67) activated protein C, protease activated receptor 1 and neuroprotection; 159–169, 2018, with permission from the American Society of Hematology. **(A)** Anticoagulant activity of APC involves the proteolytic inactivation of factors Va and VIIIa on membrane surfaces containing phospholipids that are derived from cells, platelets, lipoproteins, or cellular microparticles. The irreversible inactivation of factors Va and VIIIa to yield inactive factors Vi and VIIIi by APC is accelerated by a variety of lipid and protein cofactors (e.g., glucosyl ceramide, protein S, etc). **(B)** Beneficial direct effects of APC on cells require the EPCR and PAR-1. One distinction between pro-inflammatory thrombin signaling and cytoprotective APC signaling is the localization of APC signaling in the caveolin-1–rich microdomains (caveolae). **(C)** Neuroprotective mechanisms for APC effects on cells may also involve other receptors including PAR-3. APC-initiated signaling effects on cells can include anti-apoptotic activities, anti-inflammatory activities, inhibition of the inflammasome, stabilization of endothelial barrier functions, including the BBB, and neurogenesis. **(D)** The polypeptide structure of APC comprises an N-terminal GLA domain (green) that binds to negatively charged lipids and EPCR, 2 EGF-like domains (light blue and dark blue), and the protease domain containing the active site triad of serine, histidine, and aspartic acid residues (red). Four glycosylation sites are indicated by gray-shaded moieties. Substrate selectivity of this protease is determined by interactions between the targeted substrates and the active site and also by multiple unique binding exosites on APC that vary for different substrates. The protease domain space–filled model (see insert in D) highlights in the yellow box 3 positively charged lysine (K) residues within the so-called 37 loop (KKK 191–193), which is an exosite for APC's recognition of factors Va and VIIIa. Mutation of these 3 lysine residues to alanine (3K3A-APC) reduces APC's anticoagulant activity by >90% but does not affect its interactions with the cytoprotective substrates, PAR-1, PAR-3, or its other known cell-signaling receptors. Thus, 3K3A-APC is very “signaling-selective”.

The anticoagulant activity of APC is independent of its direct cellular effects and is mediated by irreversible proteolytic degradation of factors Va and VIIIa with contributions by other cofactors. Its cytoprotective cell-signaling activities require multiple cell-surface receptors and, in most cases, proteolytic activation of PAR-1 (Figure 1) (51, 59, 65–68).

**Figure 2:**
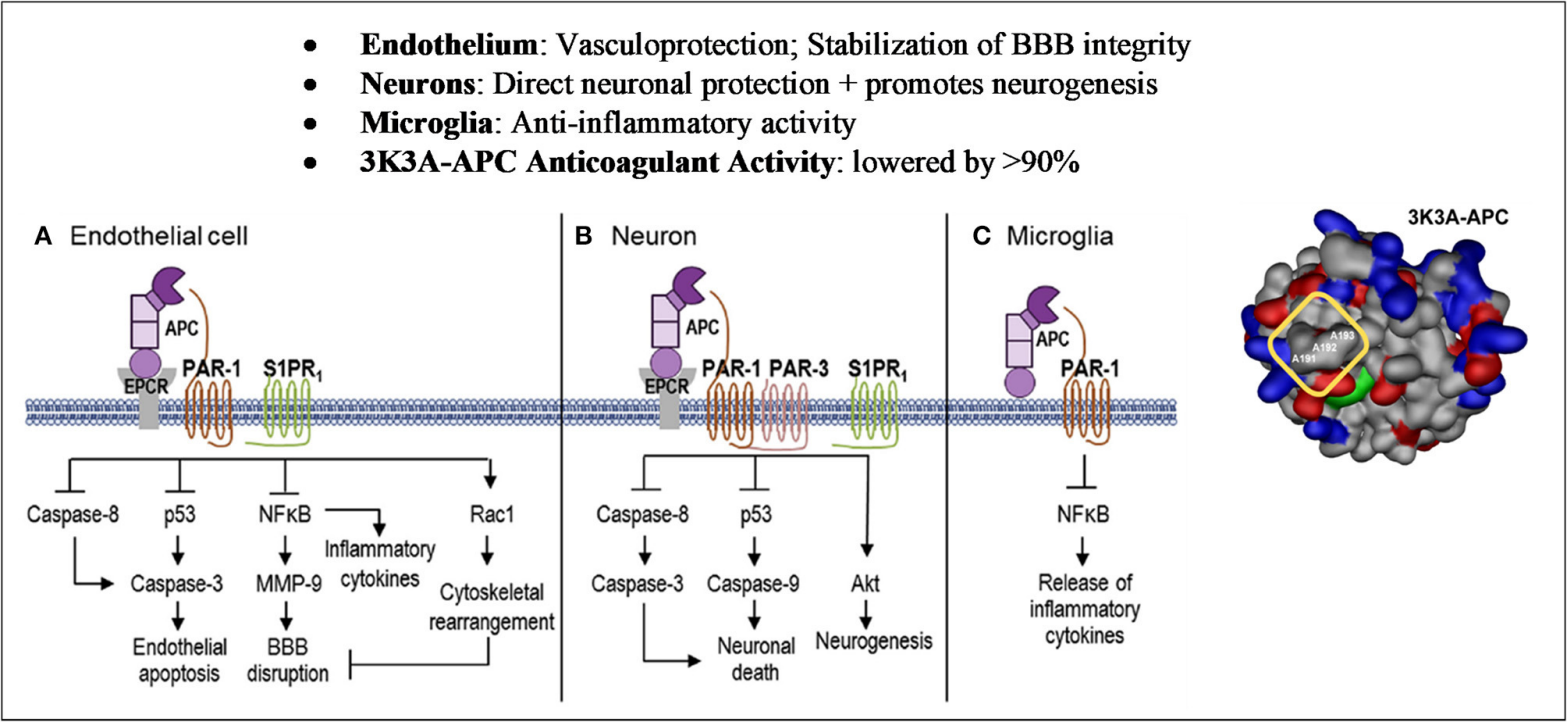
Cell-Specific APC protective signaling pathways. Akt, protein kinase B; APC, activated protein C; BBB, blood–brain barrier; EPCR, endothelial protein C receptor; MMP, matrix metallopeptidase; NFkB, nuclear factor kappa-light-chain-enhancer of activated B cells; PAR, protease-activated receptor; Rac1, Ras-related C3 botulinum toxin substrate 1; S1PR1, sphingosine 1-phosphate receptor 1. Reprinted from neuropharmacology, vol. 134, Amar et al. (69) can adjunctive therapies augment the efficacy of endovascular thrombolysis? A potential role for activated protein C, 293–301, 2018, with permission from Elsevier. 3D structure reprinted from blood, vol. 132(2), Griffin et al. (67) activated protein C, protease activated receptor 1 and neuroprotection; 159–169, 2018, with permission from the American Society of Hematology. **(A)** In endothelial cells, APC helps to seal the BBB and is vasculoprotective. APC/EPCR activates PAR-1 and inhibits caspase-8 activation of caspase-3, thereby limiting the extrinsic apoptotic pathway in endothelium. APC/EPCR-dependent PAR-1 activation suppresses the pro-apoptotic p53 transcription factor inhibiting caspase-3 activation blocking the intrinsic apoptotic pathway. Also, APC suppresses the NFkB-dependent transcriptional activation of MMP-9, thereby blocking degradation of the BBB basement membrane. Furthermore, APC blocks the expression of pro-inflammatory cytokines, limiting inflammation by controlling NFkB nuclear translocation. APC's cytoprotective effects on endothelial cells require EPCR and PAR-1 to cross-activate S1PR1. Cross-activation of S1PR1 activates Rac1, leading to stabilization of the BBB cytoskeleton, thereby supporting the integrity of the BBB. **(B)** In neurons, APC/EPCR is cytoprotective *via* PAR-1 and PAR-3, which inhibits caspase-8 upstream of caspase-3 and thereby limits the extrinsic apoptotic pathway. Also, an APC-PAR-1-PAR-3 pathway blocks p53 activation in injured neurons, thereby blocking the caspase-9-dependent intrinsic apoptotic pathway. Furthermore, APC promotes neurogenesis *via* a PAR-1-PAR-3-S1PR1-Akt pathway. **(C)** APC's inhibition of NFkB-dependent transcriptional expression of different pro-inflammatory cytokines suppresses microglial activation. Suppression of NLRP3 inflammasome development by APC is another activity but is not shown in this figure.

The cellular signaling by APC gives rise to cytoprotective alterations in gene expression profiles resulting in multiple cytoprotective actions due to anti-inflammatory and anti-apoptotic activities, as well as a reduction of endothelial barrier disruption (Figures 1, 2) (70–74). APC crosses the blood brain barrier *via* an active transport mechanism (75).

**Figure 3:**
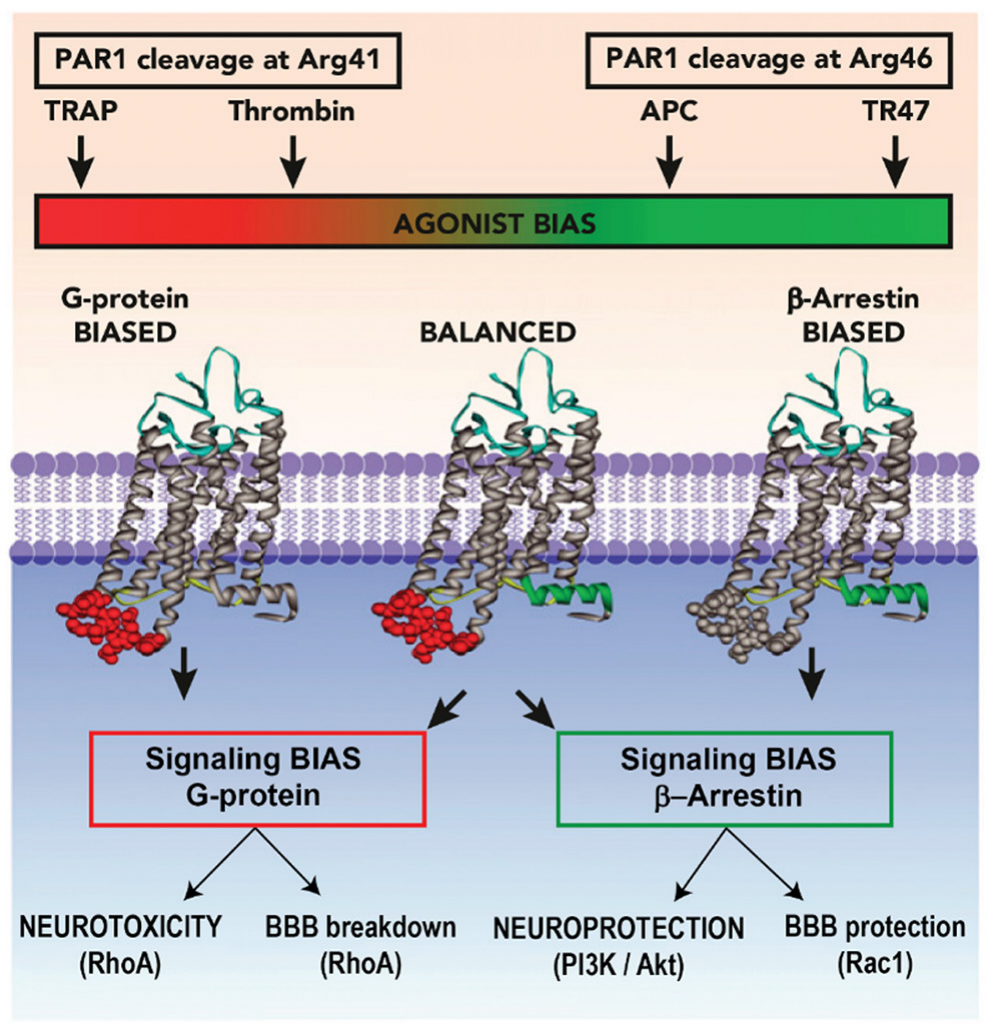
Biased Agonism of PAR-1 by APC. Akt, protein kinase B; APC, activated protein C; BBB, blood–brain barrier; PAR, protease-activated receptor; P13K, phosphoinositide 3-kinase; Rac, Ras-related C3 botulinum toxin substrate; RhoA, ras homolog gene family member A; TRAP, thrombin-receptor activated peptide. Reprinted from Blood, vol. 120(26), Mosnier et al. (68) biased agonism of protease-activated receptor 1 by activated protein C caused by noncanonical cleavage at Arg46; 5237–5246, 2012, with permission from the American Society of Hematology. Activation of PAR-1 by APC and its cytoprotective analogs involves cleavage of PAR-1 N-terminal domain at Arg46, which reveals a tethered ligand peptide that begins at Asn47 causing APC's biased, β-arrestin-2-dependent cytoprotective signaling. Activation of PAR-1 by thrombin involves cleavage at Arg41, which generates a tethered ligand that begins at Thr42, initiating cytotoxic effects *via* G-protein-dependent signaling causing human platelet activation, inflammatory changes, vascular leakage, and CNS toxicity.

For 3K3A-APC cytoprotective actions in murine preclinical ischemic stroke studies, not only is PAR-1 required but also the arginine 46 residue in PAR-1. The requirement for arginine 46 strongly supports the concept that APC cytoprotection requires “biased” signaling initiated by the G-protein coupled receptor PAR-1 (50, 68). Activation of PAR-1 by APC occurs after proteolysis of the PAR-1 extracellular N-terminal domain at arginine 46, producing a tethered ligand peptide that begins at asparagine 47 causing APC biased, β-arrestin-2-dependent cytoprotective signaling (Figure 3) (50, 68, 76–79). In contrast, activation of PAR-1 by thrombin involves cleavage at arginine 16, which generates thrombin-receptor activated peptide, a tethered ligand peptide that begins at residue 42, initiating cytotoxic effects *via* G-protein-dependent signaling and causing human platelet activation, pro-inflammatory changes, endothelial vascular leakage and CNS toxicity (67, 68, 80). PAR-1-tethered ligand peptides beginning at asparagine 47, but not those beginning at amino acid 42, exert cytoprotective effects (Figure 3) (68). Similarly, APC activates human PAR-3 by non-canonical cleavage at arginine 41, whereas thrombin cleaves PAR-3 at lysine 38 (81). PAR-3-tethered ligand peptides beginning at amino acid 42, but not those beginning at amino acid 39, exert cytoprotective effects (82), suggesting that human PAR-3 cleavage at arginine 41 by APC causes cytoprotection, whereas PAR-3 cleavage at lysine 38 initiates thrombin-like cytotoxic pro-inflammatory effects (82).

## Studies With the APC Analog 3K3A-APC

Signaling-selective APC analogs, such as 3K3A-APC, were engineered to retain normal cell-signaling activity (50–53) but to have greatly diminished anticoagulant activity (54), thereby reducing *in vivo* risk for bleeding compared with wildtype APC (55–57). In *in vitro* assays, 3K3A-APC retains the cytoprotective activity of recombinant wildtype APC but has <10% of its anticoagulant activity (e.g., see Table 3) (54).

**Table 3:** Cytoprotective and anticoagulant activity of recombinant wildtype APC vs. 3K3A-APC.

**APC type**	**Anticoagulant activity (% rwt)^a^**	**Cytoprotective activity (% rwt)^b^**	**Cytoprotective to anticoagulant ratio**
Recombinant wildtype APC	100	100	1.0
3K3A-APC	4.6^c^	114^c^	25

*APC, activated protein C; rwt, recombinant wildtype*.

a *Based on the activated partial thromboplastin time dose-response*.

b *Derived from the concentrations of APC required for half-maximal inhibition of apoptosis induced by the protein kinase inhibitor, staurosporine*.

c *From Mosnier et al*. *(**54**)*.

*Numbers for recombinant wildtype APC are definitional and represent the standard*.

The effects of 3K3A-APC on the fibrinolytic activity of tPA has also been studied *in vitro*; no statistically significant effects were noted when rt-PA was applied to induce clot lysis in the presence of either wildtype APC or 3K3A-APC (83).

3K3A-APC has beneficial effects in rodent models of stroke (50, 55–57, 72, 84–89), brain trauma (90, 91), amyotrophic lateral sclerosis (92–94), multiple sclerosis (95) and Alzheimer's disease (96), as well as ischemic injury of heart, kidney or liver, organ transplant, total body radiation, diabetes, sepsis, and wound healing (59, 67). In the CNS, PAR-1 and PAR-3 are both necessary for neuronal protection by APC (84, 85, 92), PAR-1 and endothelial protein C receptor for vasculoprotection and stabilization of the blood–brain barrier (4, 67, 69, 71–73, 84, 85, 97, 98), and PAR-1 for suppression of microglia activation and anti-inflammatory activity.^(4, 66, 91, 94)^ The extensive preclinical studies of the cytoprotective actions of APC and 3K3A-APC have been summarized in several reviews (Figure 2) (4, 59, 65–67, 69, 99).

In studies with human progenitor and fetal neural cells, 3K3A-APC promoted neurogenesis *in vitro* (52) as well as *in vivo* using a mouse middle cerebral artery occlusion (MCAO) stroke model (57).

3K3A-APC acts synergistically with tPA in both mouse and rat stroke models (55). tPA alone or in combination with 3K3A-APC, was administered 4 h after MCAO, followed by 3K3A-APC for 3–4 consecutive days afterward. In this delayed treatment paradigm, tPA alone had no beneficial effects on infarct volume, or behavior (neurological score, foot-fault, forelimb asymmetry, adhesive removal) compared with controls. In contrast, the combination of tPA plus 3K3A-APC as compared with control significantly reduced infarct volume at 24 h (65% reduction) and at 7 days (63% reduction) following MCAO in mice and at 7 days (52% reduction) after embolic stroke in rats (*p* < 0.05). Furthermore, the combination significantly improved behavioral outcomes and eliminated tPA-related intracerebral microhemorrhages (*p* < 0.01–0.05).

**Figure 4:**
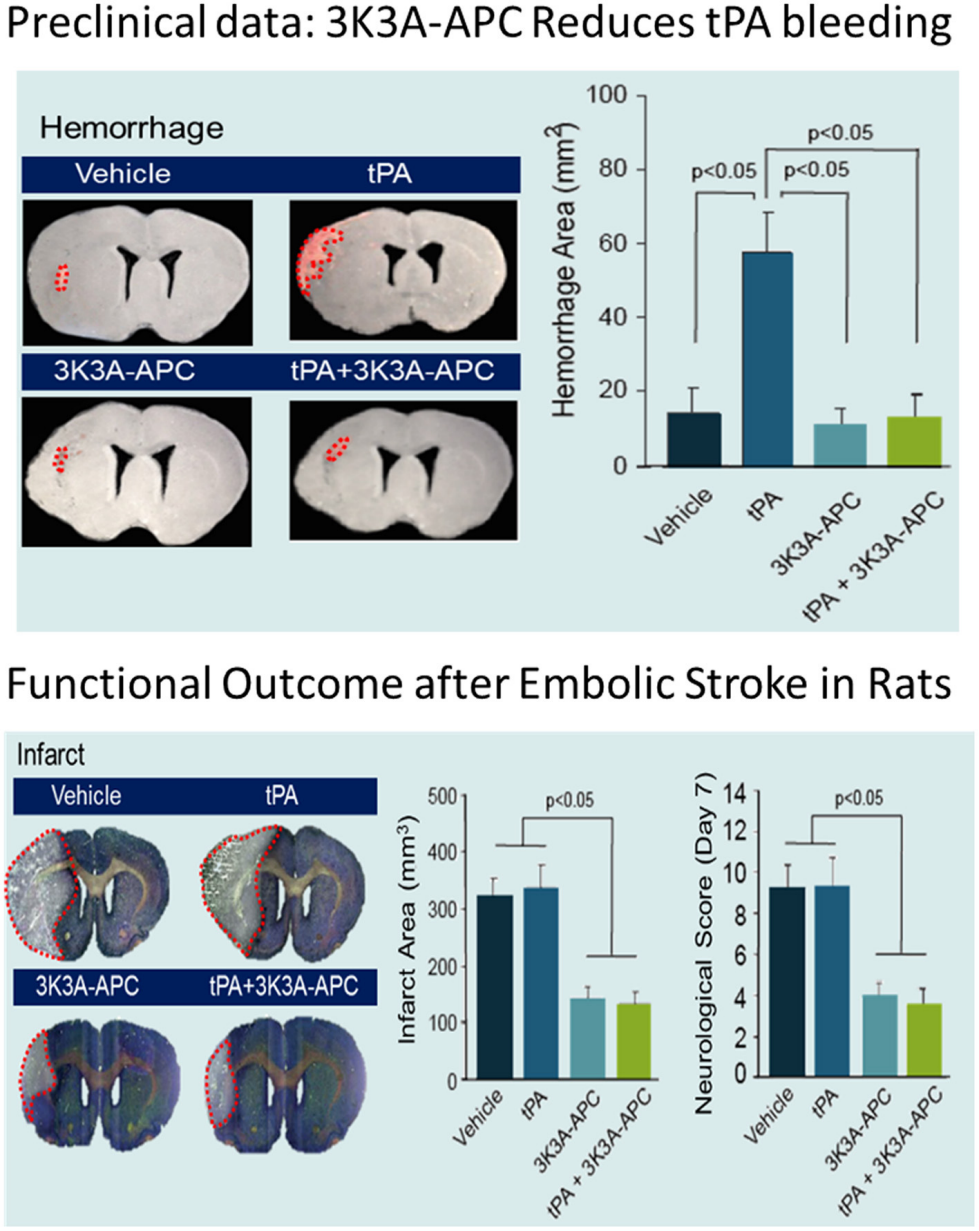
Effects of 3K3A-APC and 3K3A-APC combined with tPA on hemorrhage (upper panel) and neuropathological (hematoxylin and eosin staining; infarct volume) and neurological (neurological score) outcomes (lower panel) in young male spontaneously hypertensive rats within 7 days after embolic stroke. APC, activated protein C; SD, standard deviation; tPA, tissue plasminogen activator. Reprinted from Stroke, vol. 44(12), Wang et al. (56) activated protein C analog protects from ischemic stroke and extends the therapeutic window of tissue-type plasminogen activator in aged female mice and hypertensive rats, 3529–3536, 2013, with permission from Wolters Kluwer Health, Inc. 3K3A-APC and tPA were administered 4 h after embolic stroke. 3K3A-APC was administered for 3 consecutive days afterward. Mean + SD, *N* = 8–9 rats per group.

These positive effects of 3K3A-APC extend to elderly animals and animals with comorbidities such as might be seen in the target patient population of this study. 3K3A-APC alone or with tPA was given 4 h after transient MCAO in aged female mice and 4 h after embolic stroke in spontaneously hypertensive rats (56). 3K3A-APC was then administered from 3 to 7 days afterward. Assessments included neurological scores, foot-fault, forelimb asymmetry, and adhesive removal. In both models, tPA alone given 4 h after stroke had no effect on infarct volume or behavior. Treatment with 3K3A-APC alone or 3K3A-APC in combination with tPA reduced the infarct volume determined at 7 days by 62–66% (MCAO in aged mice) and 50–53%, (embolic stroke in spontaneously hypertensive rats), as well as improved behavior (*p* < 0.05) and significantly reduced tPA-induced intracerebral microhemorrhages (Figure 4).

Overall, in preclinical studies, 3K3A-APC appears to have a reduced risk for bleeding and provides at least equivalent if not greater cytoprotection compared with recombinant wildtype APC in mouse models of stroke. When 3K3A-APC is combined with tPA, infarct volumes are reduced and intracerebral microhemorrhages are greatly reduced and/or eliminated. At the same time, behavioral outcomes in both mouse and rat models of stroke are improved. Furthermore, 3K3A-APC expands the tPA therapeutic window, supporting further development of tPA and 3K3A-APC combination therapy (69, 99). Of note, the transient suture model of stroke that has been used in several of these studies closely mimics the clinical procedure of thrombectomy as recently reviewed (69, 99). This implies the combination of thrombectomy and 3K3A-APC for focal ischemic stroke in humans may be efficacious.

Currently, 3K3A-APC is in clinical development for stroke therapy and other indications (5).

## Need for a Stroke Therapy With Decreased Bleeding

As previously mentioned, bleeding is a risk following stroke therapies. In a randomized, controlled trial in patients who had had an arterial occlusion, the control group received standard care alone (including the use of tPA) and the thrombectomy group received mechanical thrombectomy in addition to standard care (100). AICH was more common in the thrombectomy group (51%) compared with the control group (25%).

Although mechanical thrombectomy is associated with greater bleeding and despite tPA being widely available in the United States, it is currently estimated that only 10–20% of stroke patients get tPA treatment (21, 22). According to von Kummer in 2002 (101): “The risk of brain hemorrhage is the main argument of the European authorities not to approve rt-PA, and the fear of hurting patients with rt-PA explains some of its limited use in North America. The common argument is, ‘Treatment with rt-PA may have some beneficial effect, but that is traded off by a considerable risk of symptomatic hemorrhage.’”

**Figure 5:**
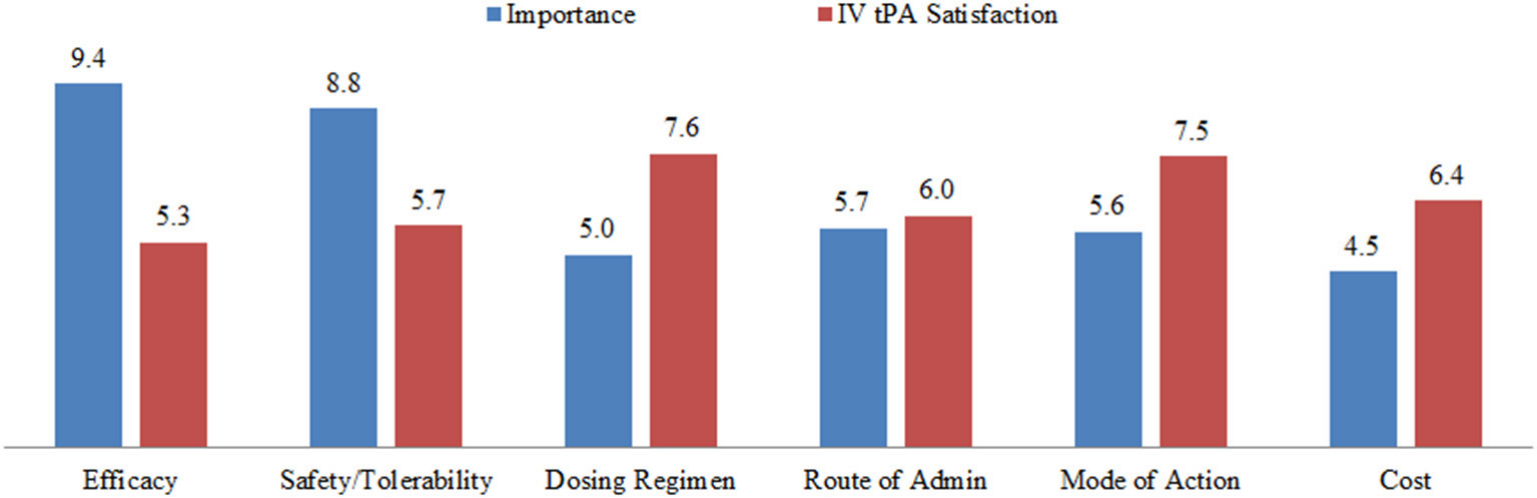
Unmet medical needs concerning physician satisfaction with tPA therapy in the United States and Europe. IV, intravenous; tPA, tissue plasminogen activator. The terms in the figure were provided to the physician interviewees without further definitions, and interviewees were asked to rank each term on a 10-point scale. For importance: 0 = unimportant and 10 = essential. For satisfaction: 0 = fully unsatisfactory and 10 = entirely satisfactory. The numbers are mean responses of the 32 interviewees.

Fear of thrombolytic-related hemorrhage influences physicians away from treating stroke (102). To quantify the effect on physicians' prescribing behavior from fear that tPA will cause intracerebral bleeding, a biopharmaceutical company obtained focus group and survey data from a private polling service for its developmental therapy for stroke, 3K3A-APC. The market research firm interviewed 34 key opinion leaders and high-volume practitioners who were practicing stroke specialists, split evenly between the United States and Europe. The majority of interviewees were neurologists who routinely treat stroke patients at a comprehensive stroke center. Each interviewee received an honorarium for his or her time. The interviewees remained anonymous to the company and to each other. They were interviewed one-on-one over the telephone in November, 2014, using a formatted target product profile and standardized questionnaire. The interviews were digitally recorded for subsequent data capture and aggregation. See Table 4 and Figure 5.

**Table 4:** Market research survey data from stroke specialists about stroke therapies.

**Question**	**Unites States neurologists**	**Unites States ER physicians**	**Unites States total**	**Europe total**	**Total**
What percentage of patients in your personal practice who are eligible for tPA receive tPA?	84%	54%	79%	87%	83%
What percentage of patients in your geographic area currently receiving tPA would receive 3K3A-APC?	93%	98%	94%	81%	88%
What percentage of patients in your personal practice currently not receiving tPA would receive the combination of tPA + 3K3A-APC were it available?	8%	13%	9%	5%	7%
What percentage of patients in your nation currently not receiving tPA would receive the combination of tPA + 3K3A-APC were it available?	17%	13%	16%	13%	15%

*ER, emergency room; tPA, tissue plasminogen activator*.

The interviews provided insights into physicians' perceptions of the relative importance of several aspects of tPA treatment, compared with their perceived satisfaction with the therapy (Figure 5). The data revealed that physicians perceive the “efficacy” of tPA to be very important (9.4/10) but not fully satisfactory (5.3/10). In contrast, the cost of the treatment was rated less important (4.5/10). Of most relevance to the present discussion, the “safety” of the drug was perceived to be very important, and rated an 8.8/10, but physicians are not satisfied with the current safety profile, giving a rating of 5.7/10. These data suggest that physicians perceive there to be a critical need for improving the safety of tPA for acute ischemic stroke.

Approximately 20% of interviewed physicians' patients eligible for intravenous (IV) tPA were not being prescribed IV tPA (Table 4) because of patient, family, or physician assessment that bleeding risk outweighed benefit (Table 4). However, the percentage of eligible patients not administered tPA was much higher in the emergency room setting (46%) relative to stroke centers (16%) (Table 4).

Physician responses suggest ~90% of patients currently being prescribed IV tPA would also be prescribed 3K3A-APC (Table 4); according to the interviewers, many physicians stated it would be a requirement in their opinion to prescribe 3K3A-APC to those patients who received IV tPA.

## Conclusion

Hemorrhagic transformation after ischemic stroke—even if labeled “asymptomatic”—may lead to long term disability and cognitive impairment. Fear of hemorrhagic transformation leads some physicians to hesitate to use indicated recanalization therapies. The PAR-1 acting agent, 3K3A-APC, reduces hemorrhagic transformation, and in animal models appears to improve long term outcomes after ischemic stroke. Agents that reduce hemorrhagic transformation may lead to wider acceptance of recanalization therapies and improved long-term outcome for ischemic stroke patients.

## Author Contributions

All authors contributed to the creation and revision of the manuscript, as well as read and approved the submitted version.

## Conflict of Interest

KP is employed by ZZ Biotech. JG, HL, JM, and BZ are paid consultants to ZZ Biotech. PL has received research funding from NIH to conduct trials of 3K3A-APC; received royalties from sales of the book Thrombolytic Therapy for Acute Ischemic Stroke, 3rd Edition; and received fees for occasional expert witness testimony. The Scripps Research Institute has intellectual property related to the topic of this review.

The remaining author declares that the research was conducted in the absence of any commercial or financial relationships that could be construed as a potential conflict of interest.
